# Impact of early cART on HIV blood and semen compartments at the time of primary infection

**DOI:** 10.1371/journal.pone.0180191

**Published:** 2017-07-14

**Authors:** Antoine Chéret, Christine Durier, Adeline Mélard, Mickaël Ploquin, Julia Heitzmann, Camille Lécuroux, Véronique Avettand-Fenoël, Ludivine David, Gilles Pialoux, Jean-Marie Chennebault, Michaela Müller-Trutwin, Cécile Goujard, Christine Rouzioux, Laurence Meyer, on behalf of the ANRS OPTIPRIM study group

**Affiliations:** 1 Internal Medicine Unit, Bicêtre Hospital, APHP, Le Kremlin-Bicêtre, France; 2 EA 7327 Paris Descartes University, Paris, France; 3 INSERM SC10-US19, Villejuif, France; 4 Virology Laboratory, CHU Necker, APHP, Paris, France; 5 Institute Pasteur, HIV, Inflammation and Persistence Unit, Paris, France; 6 INSERM U 1184, Paris Sud University, Bicêtre Hospital, APHP, Le Kremlin Bicêtre, France; 7 Infectious Diseases Department, Tenon Hospital, APHP, Paris, France; 8 Infectious Diseases Department, Angers Hospital, Angers, France; 9 INSERM, CESP U1018, Université Paris Sud, Université Paris Saclay, Faculté de Médecine Paris-Sud, Service d’Epidémiologie et de Santé Publique, AP-HP, Hôpital Bicêtre, Le Kremlin-Bicêtre, France; University of Pittsburgh Centre for Vaccine Research, UNITED STATES

## Abstract

**Background:**

HIV-infected cells in semen facilitate viral transmission. We studied the establishment of HIV reservoirs in semen and blood during PHI, along with systemic immune activation and the impact of early cART.

**Methods:**

Patients in the ANRS-147-OPTIPRIM trial received two years of early cART. Nineteen patients of the trial were analyzed, out of which 8 had acute PHI (WB ≤1 Ab). We quantified total cell-associated (ca) HIV-DNA in blood and semen and HIV-RNA in blood and semen plasma samples, collected during PHI and at 24 months of treatment.

**Results:**

At enrollment, HIV-RNA load was higher in blood than in semen (median 5.66 vs 4.22 log10 cp/mL, p<0.0001). Semen HIV-RNA load correlated strongly with blood HIV-RNA load (r = 0.81, p = 0.02, the CD4 cell count (r = -0.98, p<0.0001), and the CD4/CD8 ratio (r = -0.85, p<0.01) in acute infection but not in later stages of PHI. Median blood and seminal cellular HIV-DNA levels were 3.59 and 0.31 log_10_cp/10^6^ cells, respectively. HIV-DNA load peaked in semen later than in blood and then correlated with blood IP10 level (r = 0.62, p = 0.04). HIV-RNA was undetectable in blood and semen after two years of effective cART. Semen HIV-DNA load declined similarly, except in one patient who had persistently high IP-10 and IL-6 levels and used recreational drugs.

**Conclusions:**

HIV reservoir cells are found in semen during PHI, with gradual compartmentalization. Its size was linked to the plasma IP-10 level. Early treatment purges both the virus and infected cells, reducing the high risk of transmission during PHI.

**Clinical trials registration:**

NCT01033760

## Introduction

Viral reservoirs are established during primary HIV infection (PHI) [1], a transient stage associated with a high risk of viral transmission and responsible for up to 50% of all new infections in some areas [2, 3]. Newly infected patients are a major source of new infections, leading to clusters of transmission [4]. The risk of transmission has been estimated to be 26 times higher during PHI than during the chronic stage [5]. The risk of sexual HIV-1 transmission correlates with HIV-1 RNA load in genital secretions, which contain both free virions and infected cells [6]. Phylogenetic analysis shows different viral quasi-species in blood and semen, suggesting that the genital tract is a distinct compartment [7].

During PHI, proinflammatory cytokine and adaptive cytokine levels are elevated and linked to plasma viremia [8]. Profound dysregulation of inflammatory cytokine networks in blood and semen, creating a proinflammatory environment, may facilitate HIV-1 replication and transmission [9, 10].

Total HIV-1 DNA in PBMC is associated with markers of immune activation during both chronic HIV infection [11] and PHI [12], and is associated with HIV shedding in semen despite treatment [13]. HIV infected genital cells may be protected from environmental factors and participate in cell-cell transmission via virologic synapses. This neglected transmission pathway might be more efficient than that involving cell-free HIV [14].

Combined antiretroviral therapy (cART) reduces not only viremia but also genital HIV-1 load in chronically infected patients, thereby reducing the risk of transmission [13]. The efficacy of cART initiated during PHI on HIV genital shedding has rarely been studied [15–17], and there are no data on the genital HIV reservoir at this stage. cART initiated during PHI may not have the same impact as cART initiated in the chronic phase [18].

The aim of this work was to describe relation between HIV-RNA and cell-associated HIV-DNA in the genital tract with systemic inflammation during acute and recent PHI, and their dynamics over two years of early cART.

## Materials and methods

### Study design and participants

OPTIPRIM was a randomized, open-label, phase 3 trial comparing intensive cART versus standard triple-drug regimen initiated in primary HIV-1 infection (PHI) [19]. Briefly, 90 patients meeting the following criteria (warranting treatment initiation according to the 2010 French national guidelines) were enrolled in the trial: PHI with either symptoms or a CD4+ cell count below 500 cells/μL blood. PHI was defined by detectable plasma HIV-RNA and an incomplete HIV-1 western blot (four or fewer antibody bands), irrespective of ELISA status (positive or negative) and p24 antigen status (positive or negative), as documented within 8 days before enrollment.

### Procedure

We conducted an HIV reservoir substudy in which participants gave blood and semen samples at enrollment during PHI (day 0, before cART initiation) and at month 24. Twenty-one patients agreed to participate in this substudy. Because of poor on-site storage of 2 samples, 19 patients were studied, 12 in the intensive cART group and 7 in the standard triple-drug group.

The study was approved by the Sud-Méditerranée-1 Ethics Committee and by the French Health Products Safety Agency, and complied with the Helsinki Declaration. All the participants gave their written informed consent.

### Semen analysis

Semen samples were obtained by self-masturbation and collected in sterile containers at each participating center, then frozen at -80 C and sent to the Necker Hospital virology laboratory (Paris, France) for central analysis. In view of the limited volume of semen samples, HIV-DNA and HIV-RNA quantification were favored.

HIV-RNA was quantified in semen plasma with the Cobas Ampliprep Cobas Taqman assay v2 (Roche, France). The detection limit was below 100 cp/mL, except in two cases (133 and 200 cp/mL). For statistical analysis, a value of 100 copies/mL was arbitrarily attributed when semen HIV-RNA was undetectable.

Total HIV-DNA was extracted from semen cells by using the QIAamp DNA microkit (Qiagen, Courtaboeuf, France) and was quantified with an ultrasensitive real-time PCR method (Generic HIV-DNA assay, Biocentric, Bandol France) with a detection limit of five copies per PCR [20]. To standardize results, the total DNA in extracts was quantified using fluorescence readings at 260 nm (Nanodrop, Labtech,Ringmer, UK). DNA extracts were stored at -20°C. They were diluted in H2O to test 1 mg of total DNA per PCR, which was considered to be equivalent to 150,000 cells [21]. Each entire DNA extract was tested in two to four replicates. Results were reported as the number of HIV-DNA copies per 10^6^ cells.

### Blood analysis

HIV-RNA in blood plasma was quantified locally at all study-visits, by real-time PCR (Roche or Abbott) as described above for the semen.

Blood samples were centralized for total cell-associated HIV-DNA quantification. Thawed whole blood was analyzed with the same ultrasensitive real-time PCR method as described above for the semen (Generic HIV-DNA assay, Biocentric, Bandol, France) [20]. Each entire DNA extract (quantified with Nanodrop as previously described) was tested in two replicates, and the results were reported as the number of HIV-DNA copies per 10^6^ PBMC, taking into account the whole blood cell count.

Frozen blood plasma samples from the biobank were addressed to the Paris Pasteur Institute and Inserm U1184. Levels of IL-6, IP-10, sCD14 and sCD163 were measured in duplicate with specific ELISA assays (Human IL-6 Platinum ELISA, eBioscience; Human quantikine CXCL10 ELISA, R&D ELISA R&D; Human CD14 DuoSet ELISA and Human CD163 DuoSet ELISA, R&D Systems, Minneapolis, Minnesota). Samples with undetectable levels of a given analyte were arbitrarily attributed half the minimal detectable value.

### Statistical analyses

Demographic and clinical characteristics at baseline (D0, the day of cART initiation) were recorded as the median and range for continuous variables and as the frequency and percentage for categorical variables. We distinguished two time periods during PHI: "Acute" HIV infection was defined by the presence of one band or fewer on western blot, plus detectable plasma HIV-RNA, while the presence of at least two bands was considered to represent "recent" infection.

Continuous and qualitative variables were compared using the Wilcoxon and Chi-2 or Fisher's exact tests, respectively.

Changes in blood and semen HIV-DNA levels were expressed as the difference between M24 and D0, and differences were tested with the Wilcoxon sign rank test.

Spearman’s coefficient was used for correlation analyses of quantitative baseline characteristics and the distribution of HIV-RNA/DNA load in semen and blood. A correlogram was used to display Spearman correlations. Variables were ordered according to the angles formed by the first two principal components in PCA (Principal Component Analysis) with the following 10 active variables: baseline HIV-RNA and DNA load in semen and blood samples, the CD4 and CD8 cell counts and CD4/CD8 ratio, and IP-10, sCD14 and sCD163 levels. Levels of IL6 were not considered in the PCA analysis due to missing values. Then correlations in acute/recent PHI groups were obtained only for main parameters of interest (semen HIV-RNA, blood IP-10).

In all analyses nominal p-values were presented, since the analyses were mainly exploratory due to the small sample size. The indicative threshold for statistical significance was set at alpha = 5%. However, p-values for the 34 correlations between blood and semen viral loads and between these virological markers and markers of inflammation were also adjusted for multiplicity by controlling the false discovery rate.

SAS^®^ software version 9.3 was used for all analyses. The R package ‘corrgram’ was used.

This study is registered with clinicaltrials.gov (n° NCT01033760).

## Results

### Viral load in semen during PHI

The 19 patients were comparable to the overall OPTIPRIM population. Eighteen patients (95%) had symptomatic primary infection (Table 1). Western blot revealed acute infection in 8 patients (38%), a median of 21 days after the estimated date of infection, and recent infection in the other 11 patients, an estimated median of 29 days after infection. Seven subjects (38%) were infected by non-subtype B HIV-1.

**Table 1:** Demographic and baseline characteristics. Data are number (%) or median (min-max). MSM = men who have sex with men. PBMC = peripheral blood mononuclear cells. Acute HIV infection was defined by the presence of one band or fewer on HIV-1 western blot, plus detectable plasma HIV-RNA. P values: acute vs recent infection (Wilcoxon Exact test).

	Semen study N = 19	Acute N = 8	Recent N = 11	P-value
**Men**	19 (100%)	8 (100%)	11 (100%)	
**MSM**	18 (95%)	7 (88%)	11 (100%)	
**Age, years**	35 (20–59)	34 (30–55)	37 (20–59)	
**Symptomatic primary infection**	18 (95%)	8 (100%)	10 (91%)	
**Acute primary infection**	8 (42%)			
**Time between estimated time of infection and HIV diagnosis (days)**	25 (10–41)	21 (15–30)	29 (10–41)	0.065
**Seminal plasma HIV-RNA, log cp/mL**	4.22 (2.57–6.27)	4.30 (3.49–6.27)	4.22 (2.57–5.09)	0.20
**Seminal cell-associated HIV-DNA, log cp/10^6^ cells**	0.31 (0.00–3.58)	0.16 (0.00–2.52)	1.70 (0.00–3.58)	0.42
positive	10 (53%)	4 (50%)	6 (55%)	
negative	9 (47%)	4 (50%)	5 (45%)	
**Blood plasma HIV-RNA, log cp/mL**	5.66 (4.07–7.00)	5.68 (4.61–7.00)	5.24 (4.07–6.14)	0.15
**Blood cell-associated HIV-DNA, log cp/ 10^6^ PBMC**	3.59 (2.89–4.50)	3.77 (2.89–4.50)	3.45 (3.07–4.48)	0.62
**Blood HIV-RNA/HIV-DNA ratio**	1.58 (0.67–2.77)	2.05 (1.16–2.77)	1.53 (0.67–2.43)	0.21
**Semen HIV-RNA/HIV-DNA ratio**	3.29 (0.87–6.27)	3.95 (2.75–6.27)	2.31 (0.87–5.09)	0.062
**HIV-1 subtype B (vs. non–B)**	12 (63%)	5 (63%)	7 (6%)	
**CD4 count, cells per μL**	465 (163–1116)	373 (163–935)	471 (185–1116)	0.60
**CD8 count, cells per μL**	1088 (438–5148)	1211 (515–1626)	957 (438–5148)	0.90
**CD4 to CD8 ratio**	0.42 (0.14–1.18)	0.43 (0.22–0.75)	0.42 (0.14–1.18)	0.86
**IL-6, pg /mL n = 16**	1.11 (0.10–4.30)	0.46 (0.10–2.90)	1.12 (0.10–4.30)	0.46
**IP-10, pg/mL**	184.9 (93.9–1910.9)	129.5 (94.4–1910.9)	257.1(93.9–1807.3)	0.78
**sCD14, pg/mL**	2.10 (1.35–9.02)	1.88 (1.42–4.33)	2.20 (1.35–9.02)	0.72
**sCD163, pg/mL**	0.45 (0.25–1.60)	0.55 (0.37–0.75)	0.45 (0.25–1.60)	0.54

**Fig 1:**
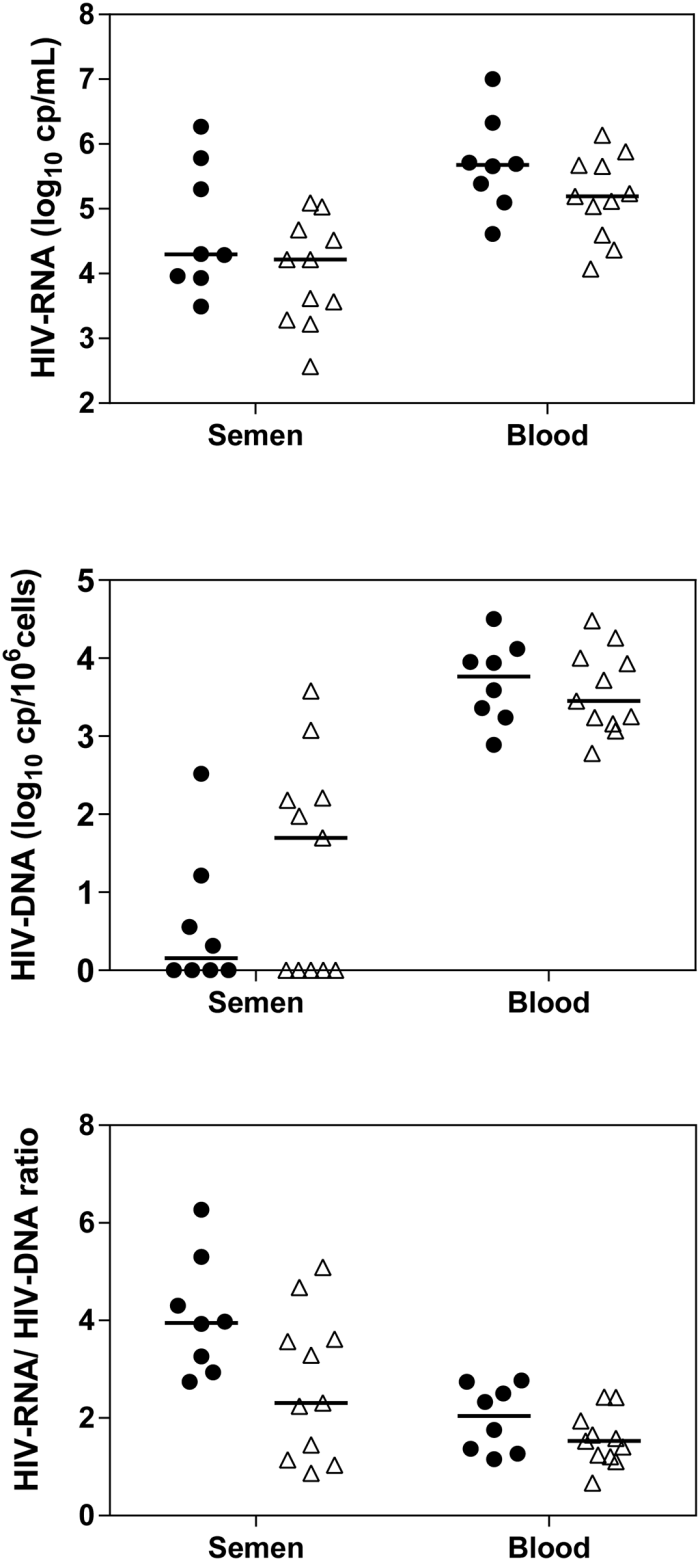
HIV-RNA and HIV-DNA load and HIV-RNA/HIV-DNA ratio during PHI before cART initiation, in blood and semen, in patients with acute infection (n = 8, black dots) and recent infection (n = 11, triangles).

During PHI, blood HIV-RNA load was higher than seminal HIV-RNA load (median 5.66 vs 4.22 log_10_ copies/mL, p< 0.0001) (Table 1 and Fig 1). Median blood-cell-associated (ca) HIV-DNA load was 3.59 log_10_cp /10^6^ PBMC. Seminal HIV-DNA was detected in only 10 of the 19 patients. Blood HIV-RNA load was higher in the patients with acute infection than in those with recent PHI infection (Fig 1), but the difference was not significant, likely owing to a lack of statistical power (5.68 vs 5.24 log cp/mL, p = 0.15) since such a difference was previously observed in a larger sample [22]. HIV-DNA load in semen tended to be higher in patients with recent infection than in those with acute infection (0.16 to 1.7 log cp/10^6^ PBMC) but not significantly, with many undetectable values (p = 0.42). The HIV-RNA/HIV-DNA ratio tended to be higher in acute infection than in recent infection, both in blood (2.1 vs 1.5, p = 0.21) and in semen (4.0 vs 2.3, p = 0.06).

### Relationship between seminal viral load and inflammation during PHI

**Fig 2:**
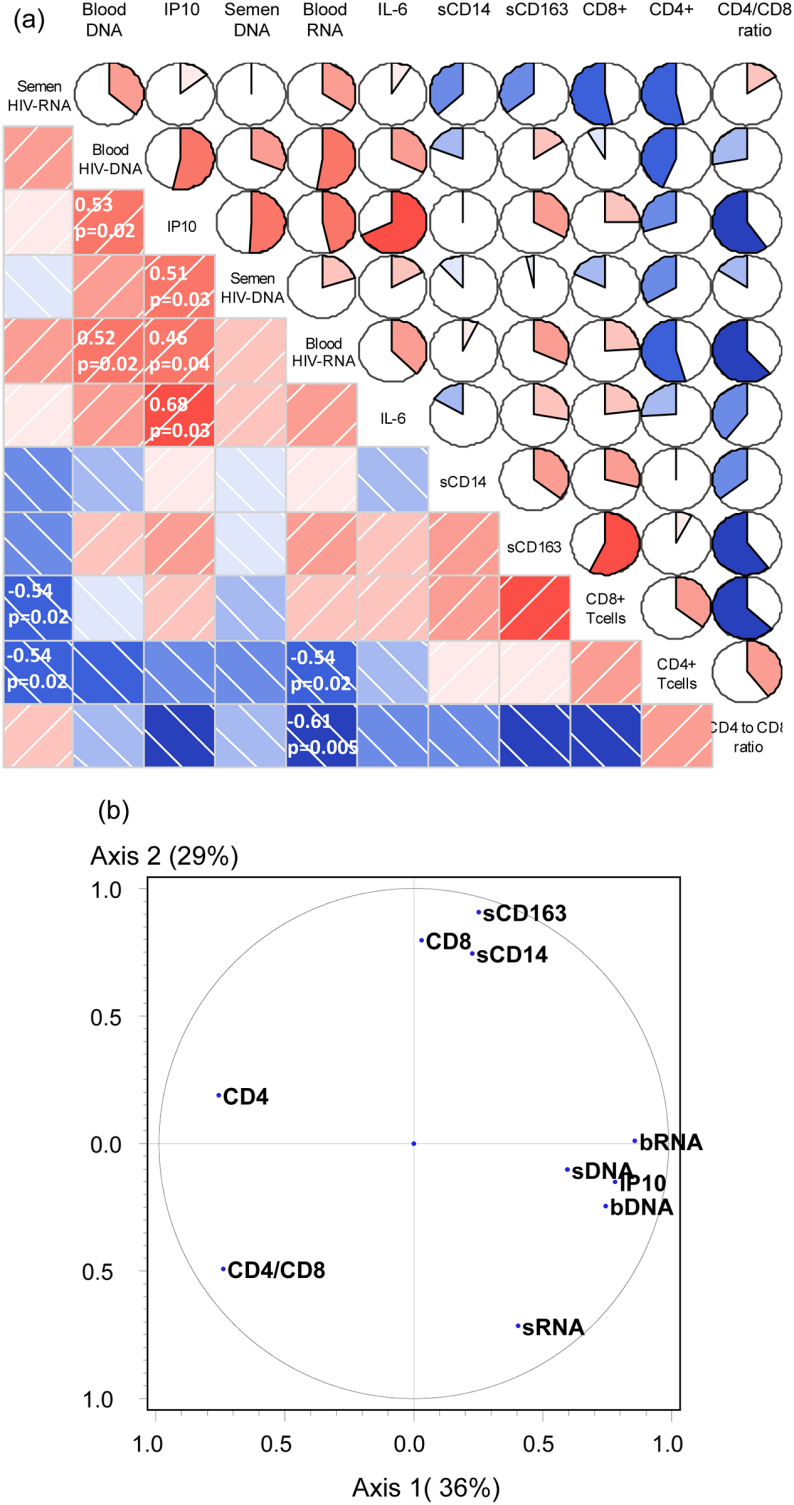
**(a)** Correlogram of baseline virological and immunological markers for 19 patients. Heatmaps and pie charts indicate associations between the variables. Red indicates a positive correlation and blue a negative correlation. The intensity and size of the colored part of pie represent the strength of the association. Spearman correlations were assumed for p values <0.05 between virological markers and immunological markers. **(b)** Circle of correlations of baseline virological and immunological markers with the first two components from Principal Component Analysis (PCA) (65% of the total variance). Blood and semen HIV-RNA and HIV-DNA are designated as follows: bRNA, bDNA, sRNA, sDNA.

The relationship between blood and semen viral loads and markers of inflammation was studied first in the entire study population (Fig 2a). Semen HIV-RNA load negatively correlated with peripheral CD4+ (r = -0.54, p = 0.018, adjusted p = 0.12) and CD8+ T cell counts (r = -0.54 p = 0.018, adjusted p = 0.12) (Fig 2a). Semen HIV-DNA load correlated positively with blood IP-10 levels (r = 0.51, p = 0.026, adjusted p = 0.12). Other markers of inflammation, including blood IL-6, sCD14 and sCD163 levels, did not correlate with virological markers.

Principal components analysis (PCA) was used to assess relationships between inflammatory markers and virological markers in blood and semen: the first PCA dimension was mainly related to virological parameters and IP-10, and to the CD4 cell count and CD4/CD8 ratio on the opposite side of the dimension, while the second dimension was associated with the CD8 T cell count and monocyte markers (sCD163, sCD14) and, to a lesser extent, semen HIV-RNA load (Fig 2b).

**Fig 3:**
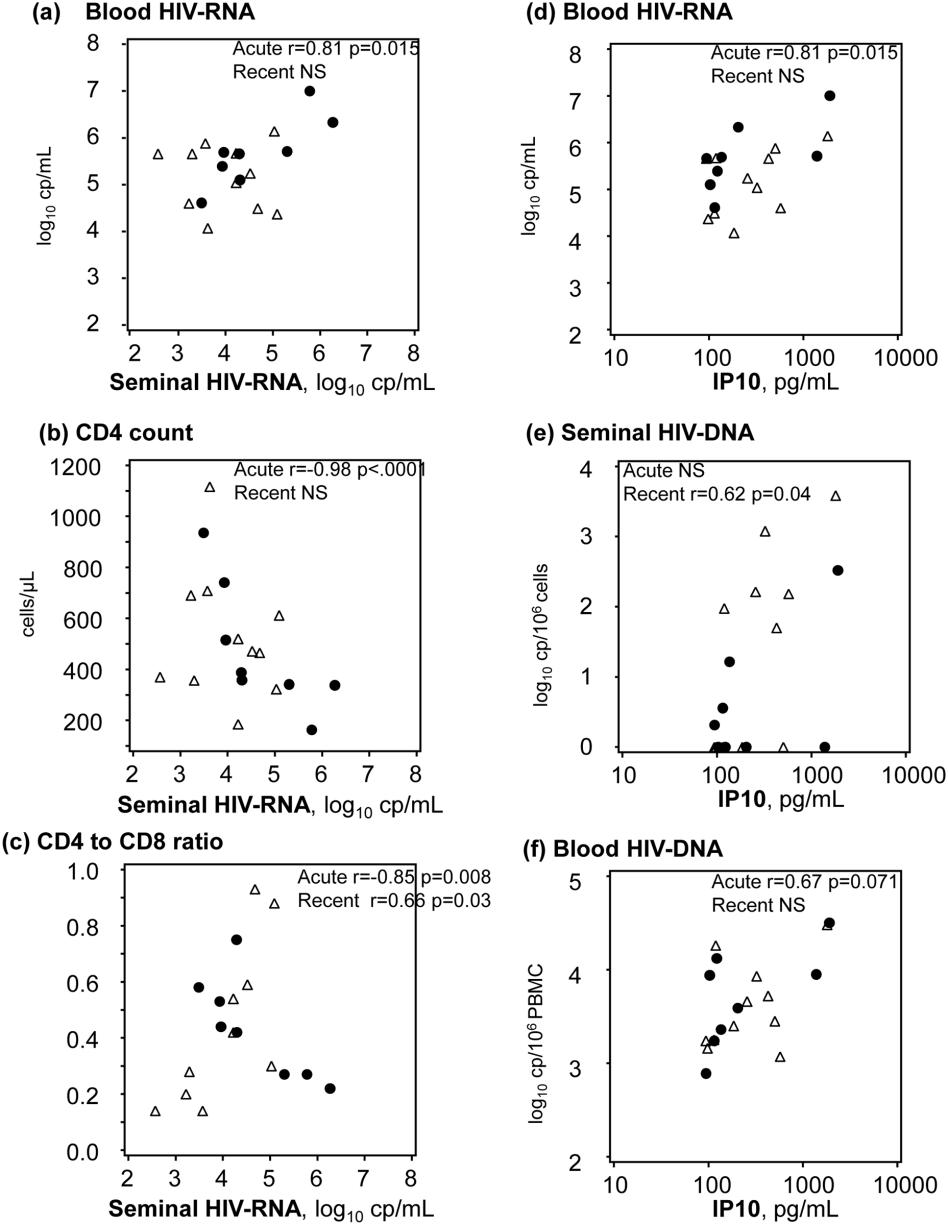
Correlations between seminal HIV-RNA and (a) blood HIV-RNA, (b) the CD4 cell count, and (c) the CD4/CD8 ratio, and between IP-10 and (d) blood HIV-RNA (e) seminal HIV-DNA, and (f) blood HIV-DNA, according to primary infection status: Acute infection (black dots), and recent infection (triangles).

Among the 8 patients with acute infection, a correlation was observed between semen and blood HIV-RNA loads (r = 0.81, p = 0.015, adjusted p = 0.10) (Fig 3a). Strong correlations were also observed between semen HIV-RNA load and the CD4+ cell count (r = -0.98, p<0.0001, adjusted p = 0.001) (Fig 3b), and the CD4/CD8 ratio (r = -0.85, p = 0.008, adjusted p = 0.10) (Fig 3c), while seminal RNA did not correlate with blood RNA and CD4 counts in recent infection. In patients with recent infection, semen HIV-RNA load correlated negatively with monocyte activation markers (CD8, sCD163, sCD14) (Parts A, B and C in S1 Fig).

Semen HIV-DNA load correlated with IP-10 levels in recently infected patients (r = 0.62, p = 0.04, adjusted p = 0.20), but not in acutely infected patients, whose semen HIV-DNA load was low (Fig 3e). IP-10 levels correlated with blood HIV-RNA and to a lesser extent blood HIV-DNA load in acutely infected patients (r = 0.81; p = 0.02; adjusted p = 0.10 and r = 0.67; p = 0.07; adjusted p = 0.34, respectively) (Fig 3d) (Fig 3f) but not in recently infected patients.

### Impact of early cART on blood and genital viral parameters after 24 months

**Fig 4:**
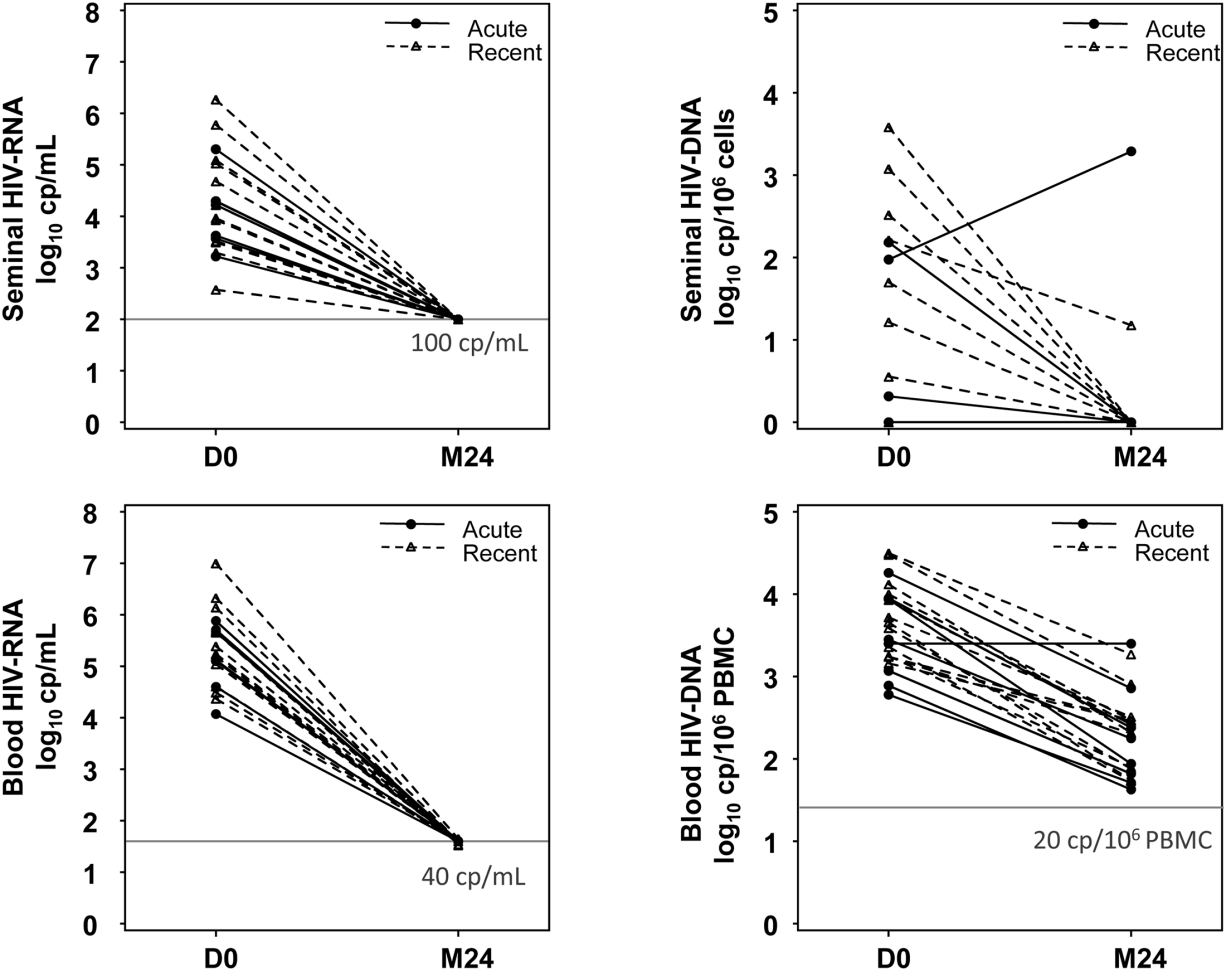
Changes in seminal and blood HIV-RNA and HIV-DNA between D0 (cART initiation during PHI) and M24 (2 years of cART). As shown on the right panel, an increase in seminal HIV-DNA load with stable blood HIV-DNA load occurred in two patients. Horizontal lines are the detection thresholds.

After two years of cART initiated during PHI, all the patients had undetectable HIV-RNA load in blood and semen (Fig 4). HIV-DNA load in PBMC fell by a median of 1.35-fold/log10 cp/10^6^ PBMC (p<0.0001): median HIV-DNA load at M24 was 2.32 log10 cp/10^6^ PBMC (range 1.63–3.40). Seminal HIV-DNA load declined similarly, by a median of 0.31 log10 cp/cells (n = 17, p = 0.019): HIV-DNA levels were undetectable at M24, except in two patients: one was at the detection limit, while the other had a clear increase of 1.3 log10 cp/cells. Of note, this latter patient also had persistently high IP-10 and IL-6 levels and reported having started to use recreational drugs a few weeks previously. No episodes of sexually transmitted infection were reported; moreover, urinary PCR for *Chlamydiae trachomatis* and *Neisseriae gonorrhoea*, and hepatitis C and syphilis serologies were negative within one month following M24.

## Discussion

This is the first quantitative and longitudinal study of the seminal viral reservoir during primary HIV infection. We found that pretreatment HIV-RNA load was high in semen during PHI (median 4.2 log/mL), albeit lower than in blood. This is consistent with previous studies in PHI [15] and of SIV infection in primates [23], and particularly with results from a study of patients with early PHI [17]. Eight of our patients were recruited at the 4^th^ G3 stage of Phanuphak,[17] while the others were recruited slightly later, corresponding to the frequent timing of PHI diagnosis in clinical practice.

Semen HIV-RNA load well correlated with blood HIV-RNA load in patients with acute infection (≤ 1 WB band) but not in those with recent infection. A similar but weak correlation was observed in the few previous studies of PHI with no distinction between acute and early patients [15]. We therefore here were able to underline the correlation between blood and semen HIV-RNA load in acute patients. We also found here a strong association, as previously described in chronic infection [22], between semen HIV-RNA load and the severity of immunodeficiency in acutely infected patients, i.e. those patients who had high blood HIV-RNA load and low CD4 cell counts. These results might be explained by genital virus replication associated with local immunomodulation, independently of viral replication in the blood compartment [24].

Seminal and blood HIV-DNA loads were high during PHI, with a trend towards a higher seminal HIV-RNA/HIV-DNA ratio in acutely infected patients than in recently infected patients. Moreover, blood HIV-DNA load tended to be lower in recently infected patients than in acutely infected patients, while it tended to be higher in semen. These results obtained for the first time about HIV-DNA in semen suggest that, during PHI, the semen reservoir is established later and more gradually than the blood reservoir, and support the hypothesis of HIV compartmentalization in the genital tract during PHI [7]. This might be explained by the gradual establishment of HIV in infected cells in acute patients, while transfer of infected cells can occur later in recent patients [25]. However, we did not have the possibility to proceed to phylogenetic analysis.

We evaluated the inflammatory status in the patients by measuring proinflammatory cytokines (IL-6, IP-10) and markers of monocyte activation (sCD14 and sCD163) in blood [26]. Blood and semen are two different compartments with respect to virological and immunological status [10], probably limiting the interaction between seminal HIV replication and plasma cytokine during PHI. Nevertheless, we found that monocyte activation markers (sCD163, sCD14) correlated negatively with semen HIV-RNA load in recently infected patients. One explanation could be that early innate immunity contributes to the control of HIV replication in semen.

Previous studies in chronically infected patients reported several strong associations between seminal HIV-RNA viral load and seminal cytokine levels, including IL-6, TNFα, IL-8 [27], IL-17,IL-5 [24], G-CSF, INFƔ [9], IL—1[28], and RANTES [29], and less associations were found with blood cytokines, namely IL-12 and INFƔ [9, 24]. Here, seminal viral ca-DNA correlated with plasma IP-10, mostly in recent infection, and not during acute infection. Apart from IP-10, there were no other positive correlation with virological parameters. Two recent studies showed that IP-10 was the only cytokine that correlated positively with HIV-RNA load at different Fiebig stages [30, 31], and the only cytokine (among 22) that differed between patients who did and did not start treatment during PHI [32]. Moreover, plasma IP-10 levels were of strong predictive value for rapid disease progression in HIV chronic infection [25]. It has been shown that IP-10 facilitates HIV infection of resting CD4+ T cells and IP10 might furthermore preferentially attract major HIV target cells (memory CD4+ T cells) to the sites of inflammation [33]. As in other studies, we found that IP-10 correlated strongly with both viral RNA and viral ca-DNA in blood [33]. Plasma IP-10 also correlated with seminal HIV-DNA, particularly among recently infected patients. Again, this may be explained by gradual HIV compartmentalization in the genital tract; this is also in line with recent evidence that IP-10 contributes to amplification of HIV reservoirs in lymphoid organs [34, 35].

Two years of early cART markedly reduced viral load (HIV-RNA and HIV-DNA) not only in blood but also in semen, adding a new argument to the benefit of early treatment at PHI [19]. There were no differences between groups at month 24, but the lack of longitudinal data to evaluate the kinetics of semen HIV-DNA and HIV-RNA levels does not allow to specify the impact of the treatment between the two groups. Surprisingly, one patient had high semen HIV-DNA load at month 24, despite persistently undetectable plasma viral load, excellent self-reported adherence and the absence of sexually transmitted infection at this time point. It is noteworthy that this patient reported cocaine use at month 22 and 23. Cocaine has been associated with immunological changes, enhanced HIV replication in vitro and in animal models [36], and permissiveness of quiescent T cells for HIV infection [37]. A recent study suggested that active cocaine use among HIV-1 patients is associated with a lack of viral suppression, independently of treatment adherence [38]. Cocaine use by this patient might thus have induced several transient viral shedding events, as reported with use of cannabis [13] that helped to replenish his genital HIV reservoir.

Our study had some limitations. In particular, the study size was small, owing to the difficulty of obtaining semen samples very early in the infection. In spite of this small sample size, interpretation of the results was based on high estimated correlations and adjusted p-values remaining generally around .10. Nevertheless these results should be interpreted with caution due to numerous variables interrogated for correlations. Moreover, principal component analysis also identified two directions, virological parameters and IP-10 opposed to CD4 T cell on one side, and CD8 T cell and monocyte markers on the other side, allowing to visualize graphically the correlations.

In addition, we had limited information on other factors that can modulate genital tract inflammation, such as herpesvirus shedding or chlamydiae and gonorrhea infection in particular at baseline which could affect immune inflammation and risk of HIV transmission [39, 40]. Finally, the semen samples were too small to explore the capacity of infected seminal cells to produce infectious virus in cell culture.

It is important to note that seminal HIV-RNA levels found in our study may not be fully representative of primary-infected patients, as 95% of the patients were symptomatic [41]. Indeed, seminal viral load might be lower in patients with asymptomatic PHI, as reported for plasma viral load [22]. In this study, we present results from blood and semen samples of patients with the potentially highest risk of viral transmission.

In conclusion, despite the small sample size, we provide the first evidence that HIV reservoir cells in semen begins to establish early during HIV infection. This seminal reservoir is established gradually, with a probable steady state link with blood IP-10 level, underlying the important role of this cytokine during PHI. Detailed phylogenetic analysis should be performed to strengthen the hypothesis regarding blood-semen compartimentalisation driven by IP-10. This time shift establishment is contemporary with high viral load in seminal plasma, implying a major risk of transmission. The presence of infected cells in semen is likely to increase the risk of HIV transmission during PHI, via cell-cell contact. We show that early treatment purges not only viral particles but also infected cells in the genital compartment.

## Supporting information

S1 Fig Correlations between seminal HIV-RNA and (a) the CD8 cell count (μL), (b) sCD163 (pg/mL) (c) sCD14 (pg/mL), according to primary infection status: Acute infection (black dots) and recent infection (triangles). (TIF)
