# Supplementary figures and images for: Impact of early cART on HIV blood and semen compartments at the time of primary infection
Source: PLoS One. 2017 Jul 14;12(7):e0180191. doi: 10.1371/journal.pone.0180191 (PMC5510829; doi:10.1371/journal.pone.0180191)

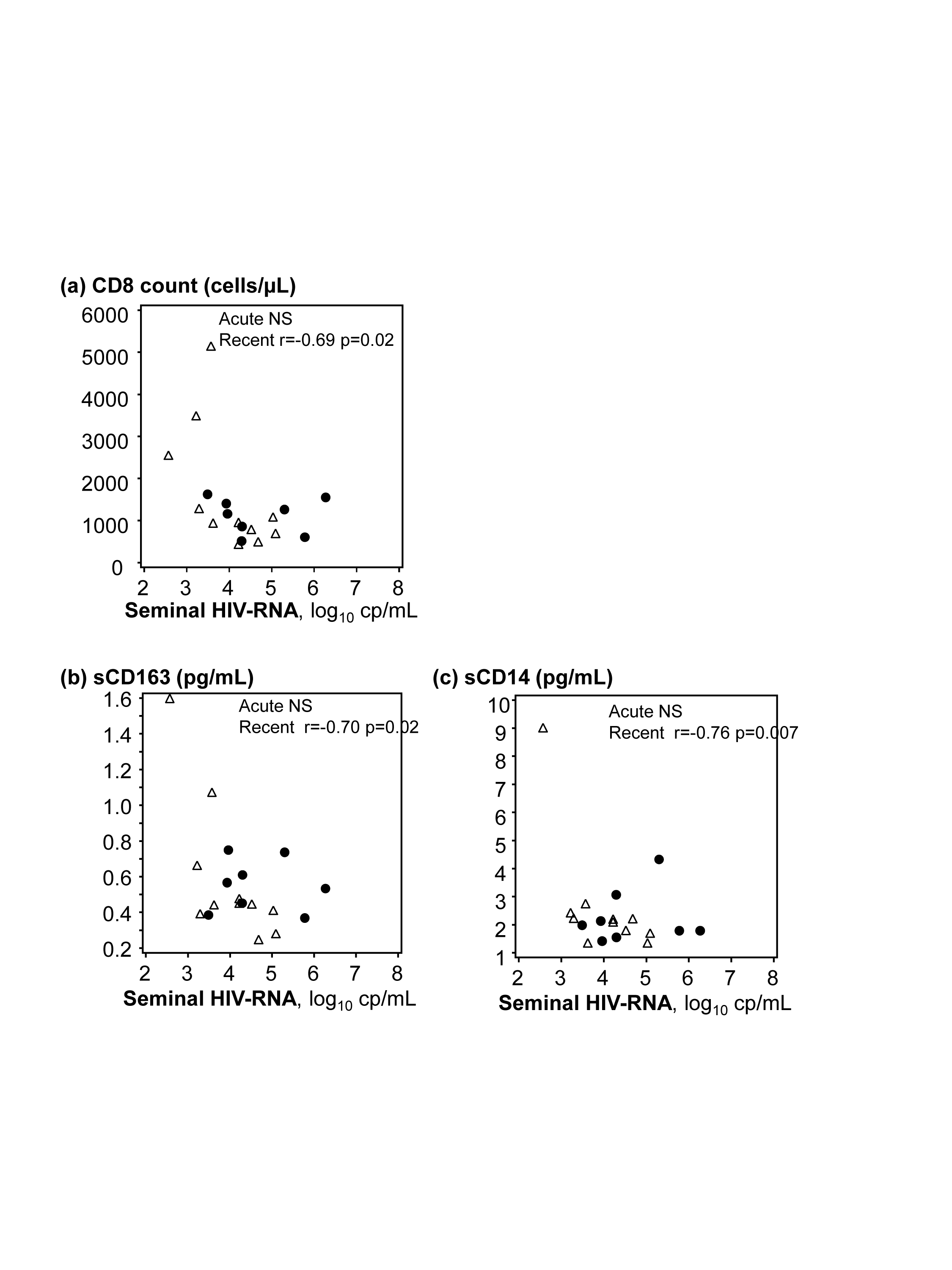

Supplement: S1 Fig — (TIF) [file pone.0180191.s001.tif]
